# Relationship Between Epilepsy and Dreaming: Current Knowledge, Hypotheses, and Perspectives

**DOI:** 10.3389/fnins.2021.717078

**Published:** 2021-09-06

**Authors:** Aurélien de la Chapelle, Birgit Frauscher, Amandine Valomon, Perrine Marie Ruby, Laure Peter-Derex

**Affiliations:** ^1^Lyon Neuroscience Research Center, CNRS UMR 5292, INSERM U1028—PAM Team, Lyon, France; ^2^Analytical Neurophysiology Lab, Montreal Neurological Institute and Hospital, McGill University, Montreal, QC, Canada; ^3^Center for Sleep Medicine and Respiratory Diseases, Lyon University Hospital, Lyon 1 University, Lyon, France

**Keywords:** sleep, epilepsy, seizures, memory, dream, emotion

## Abstract

The interactions between epilepsy and sleep are numerous and the impact of epilepsy on cognition is well documented. Epilepsy is therefore likely to influence dreaming as one sleep-related cognitive activity. The frequency of dream recall is indeed decreased in patients with epilepsy, especially in those with primary generalized seizures. The content of dreams is also disturbed in epilepsy patients, being more negative and with more familiar settings. While several confounding factors (anti-seizure medications, depression and anxiety disorders, cognitive impairment) may partly account for these changes, some observations suggest an effect of seizures themselves on dreams. Indeed, the incorporation of seizure symptoms in dream content has been described, concomitant or not with a focal epileptic discharge during sleep, suggesting that epilepsy might directly or indirectly interfere with dreaming. These observations, together with current knowledge on dream neurophysiology and the links between epilepsy and sleep, suggest that epilepsy may impact not only wake- but also sleep-related cognition.

## Introduction

Epilepsy is a neurological disease characterized by an “enduring predisposition to generate epileptic seizures,” defined as “transient occurrence of signs and/or symptoms due to abnormal excessive or synchronous neuronal activity in the brain” (Fisher et al., 2005). Epilepsy is not only characterized by seizures, but it is further complicated by significant comorbidities such as cognitive impairment and psychiatric disorders (Fisher et al., 2005; Keezer et al., 2016). The interactions between epilepsy and sleep are numerous, including the comorbidity between epilepsy and sleep disorders, the activating effect of sleep on seizures and interictal epileptic activity, and the disturbance of sleep macro- and microstructure by these abnormalities (Bazil, 2000; Van Golde et al., 2011; Peter-Derex et al., 2020; Bergmann et al., 2021). Thus, epilepsy is likely to have an impact on sleep-related cognitive activity (e.g., memory consolidation, stimuli perception/integration, dreaming).

The cognitive processes at work during dreaming currently remain imperfectly understood. According to the phenomenology of dream reports, they comprise at least sensory and associative processes, short and long term memory processes, executive and emotional processes, mind-reading, and consciousness: in other words nearly all the cognitive processes used during wakefulness (Nir and Tononi, 2010; Kahan and Laberge, 2011; Ruby, 2011, 2020; Windt et al., 2016).

Epilepsy is prone to interfere with these mechanisms in different ways. Seizures may influence dream content with possible incorporation of symptoms of diurnal or nocturnal seizures, especially when these latter occur during sleep when a dream is unfolding. Epilepsy may also modulate the ability to encode or remember dreams. Such ability is likely to be altered notably by the dysfunction of brain regions critical for the formation/recall of dream memories [medial prefrontal cortex (MPFC) and temporo-parieto-occipital junction (TPOJ)] (Solms, 2000, 2011; Eichenlaub et al., 2014b; Vallat et al., 2020) due to epileptic activity or due to the underlying neurological condition. On the contrary, it could also be promoted by increased wake after sleep onset observed in epilepsy patients (Koulack and Goodenough, 1976; Eichenlaub et al., 2014a; Vallat et al., 2017b; Sudbrack-Oliveira et al., 2019). Conversely, symptoms of daytime seizures may sometimes resemble dreaming experiences in some aspects, such as the oneiric atmosphere associated with a feeling of strangeness and familiarity, the so-called “dreamy state” (Jackson and Colman, 1898; Bancaud et al., 1994; Vignal et al., 2007), or by the feeling of experiencing or remembering a previous dream evoked during a seizure, the so-called “déjà-rêvé” (Curot et al., 2018).

In this review, we propose to discuss existing knowledge on the links between dreaming and epilepsy. In particular, we (i) provide a summary of the current understanding of the neurophysiological basis of dreaming, (ii) present the close interactions between epilepsy and sleep suggesting that epileptic activity might interfere with dreaming processes, and describe the potential relationship between seizures and dreams, both for sleep- and wake-related seizures; (iii) provide a synthesis of existing knowledge on the specificities of dreams in epilepsy patients and discuss these data in light of what is known about the determinants of dream recall and dream content in healthy subjects; and (iv) propose a perspective on the possible implications of dream disturbances in epilepsy, current research gaps and potential future developments.

## Neurophysiology of Dreaming

The definition of dreaming is still under debate (Pagel et al., 2001) but it is admitted that dreaming can be considered as a cognitive activity involving “mental imagery that consists of sensory hallucinations, emotions, story-like or dramatic progressions, and bizarreness” during sleep (Nielsen, 2000). Up to now, the brain mechanism at work during dreaming remains poorly understood due to methodological, technological and theoretical locks (Ruby, 2020). Indeed, except in specific situations, most often non-physiological [rapid-eye-movement (REM) sleep behavior disorders, lucid dreaming], only dream reports can be studied and not the dream experience itself (Guenole and Nicolas, 2010). Keeping these restrictions in mind, we present below a summary of the current knowledge and concepts regarding the neurophysiology of dreaming.

### Models of the Neurophysiological Basis of Dreaming

Based on neuropsychological, electrophysiological and functional neuroimaging studies, two main models have been proposed. A “two-generator model” [one in REM and one in non-REM (NREM) sleep] or “activation-synthesis hypothesis” posits that brain mentation during sleep would vary along three dimensions: (i) activation, which depends on the brainstem reticular activating system; (ii) input, which refers to the processing of exteroceptive or interoceptive stimuli and depends on the input-output gating and the level of activation of the sensorimotor cortex; and (iii) modulation, which refers to the neuromodulatory balance (shift from noradrenergic and serotoninergic tone in waking to cholinergic tone in REM sleep) and is involved in regulation of conscious state function and insight. These dimensions would behave differently according to vigilance states, which would explain quantitative and qualitative differences in dream reports between REM and NREM sleep (Hobson et al., 2000; Fosse et al., 2001). By contrast, a “one-generator model” proposes that dreaming would be under the control of a “trans-state” cortical and dopaminergic “dream on” mechanism involving the ventro-mesial frontal region and the TPOJ (Solms, 2000, 2011). This model would explain (1) why dreams occur not only in REM, but also in NREM sleep [50% in average and up to 90% of awakenings in NREM sleep are followed by dream reports, some of which are undistinguishable from REM dream reports (Cavallero et al., 1992; Cicogna et al., 1998; Nielsen, 2000; Wittmann et al., 2004), especially in the morning light NREM due to a circadian effect on dream recall (Cicogna et al., 1998; Chellappa and Cajochen, 2013)], (2) the increase in dream recall with increasing dopamine. It also suggests that dreaming might be facilitated in other situations such as epileptic activity and toxic/metabolic conditions (Solms, 2000, 2011).

### Experimental Results on the Neurophysiological Basis of Dreaming and Dream Recall

Several studies have demonstrated the involvement of specific brain structures in dream recall and/or production. The MPFC whose acute lesions result in a cessation of dream reports would play a central role in dreaming (Solms, 2000; Eichenlaub et al., 2014b; Vallat et al., 2018, 2020). The MPFC is part of the mesocortical dopaminergic pathways (Lena et al., 2005; Solms, 2011) and of the limbic system, which is particularly active during the “dreaming + REM sleep” state and may account for the emotional and mentalizing features of dreams (Maquet et al., 1996, 2005; Eichenlaub et al., 2014b). The TPOJ, which plays an important role in mental imagery, episodic memory and perspective taking, is also considered as a key generator of dreams (Maquet et al., 2005; Solms, 2011; Eichenlaub et al., 2014b; Vallat et al., 2020) with occipital areas being involved in the visual component of dreams (Solms, 2011). The dorsolateral prefrontal cortex (dlPFC), would rather be deactivated during dreaming, except during lucid dreaming (Dresler et al., 2012; Vallat and Ruby, 2019), accounting for the alteration of executive functions during dreaming (Maquet et al., 1996; Muzur et al., 2002; Ruby, 2011) and its reactivation would help recalling dream content at awakening (Vallat et al., 2020). It is however important to keep in mind that the memory system involved in the dreaming process may be partly distinct from the one at work during wakefulness. Indeed, dream memories and episodic memories of diurnal experiences are very rarely confused even if similar in content, and dreams can be reported in amnesic patients even with bi-hippocampal lesions (Torda, 1969). Interestingly, in contrast with the preceding results, Fell et al. (2006) findings suggested that the rhinal-hippocampal connectivity would subserve the incorporation of episodic memory content in dream content and would participate in dream recall (Fell et al., 2006).

Some other studies have investigated the neurophysiological correlates of dream recall by analyzing the brain activity preceding (or following) an awakening associated with a dream recall (Ruby, 2020). Several features have been associated with dream recall, such as a decrease in slow wave activity in posterior regions (see Ruby, 2020, for a review), local frontal activation (Siclari et al., 2018), and changes in brain functional connectivity (Nieminen et al., 2016) in the sleep just preceding awakening. Other works have suggested that brain patterns of activity, with local increase in high frequency activity recorded with high density EEG or local increase in the BOLD signal (fMRI), might be associated with specific dream contents (Horikawa et al., 2013; Siclari et al., 2017). Finally, our team (CRNL) and other colleagues have emphasized the role of awakenings in dream recall whose frequency/duration and efficiency (in terms of memory encoding) would depend on a specific activity notably in the default mode network during sleep and at awakening (Eichenlaub et al., 2014a,b; Vallat et al., 2017b, 2020; Van Wyk et al., 2019). According to these works and to the “arousal-retrieval” model (Koulack and Goodenough, 1976), dream recall would require an awakening when the short-term memory of the dream experience is still “alive” and would depend on the brain’s ability to return to a cognitive functioning closer to the waking state (Schredl et al., 2003; Ruby et al., 2021). This hypothesis which gives an important role to short-term memory in dream recall, is coherent with results showing that short-term memory [involving the prefrontal cortex, (Nee and Jonides, 2008)] is at least partially preserved in N2 and REM sleep, even if dlPFC possible deactivation during sleep may diminish its capacity and duration (Daltrozzo et al., 2012).

## Epileptic Seizures and Dreams

Epilepsy is prone to interfere in many ways with the processes involved in dreaming, especially as sleep has a strong activating effect on epileptic activity.

### Epilepsy and Sleep Interactions

Epilepsy results from the development of a neuronal hyperexcitability, due to a pathogenetic injury of various causes, which leads to recurrent focal or generalized seizures (Devinsky et al., 2018). Epilepsy and sleep interact in multiple ways (Malow, 2007; Grigg-Damberger and Foldvary-Schaefer, 2021; Moore et al., 2021). On the one hand, sleep has a modulating effect on epileptic activity; some epilepsy syndromes are characterized by seizures occurring almost exclusively during sleep, or by a major activation of inter-ictal epileptiform discharges during sleep, especially NREM sleep (Frauscher and Gotman, 2019; Nobili et al., 2020). This activating effect of NREM sleep on epilepsy is modulated by the depth of sleep: lighter stages of NREM sleep promote seizures, whereas deeper stages associated with high amplitude slow waves are more likely to enhance inter-ictal epileptiform discharges (Minecan et al., 2002; Frauscher et al., 2015). Conversely, REM sleep (particularly the phasic REM sleep microstate) has an inhibitory influence on epileptic activity potentially linked to neuronal desynchronization mediated by cholinergic neurotransmission (Ng and Pavlova, 2013; Frauscher et al., 2016). Although very rare, epileptic seizures can nonetheless be observed during REM sleep (Minecan et al., 2002; Peter-Derex et al., 2020). Chronobiological factors also influence the occurrence of seizures (Karoly et al., 2021). On the other hand, epilepsy has a disrupting effect on sleep macro- and micro-architecture. It is associated with an increase in wake after sleep onset (Sudbrack-Oliveira et al., 2019; Peter-Derex et al., 2020) but also with a micro-structural fragmentation of sleep, linked to both ictal and inter-ictal epileptic activity (Malow et al., 2000; Peter-Derex et al., 2020). Recently, it has been proposed that epileptic activity might disrupt NREM sleep oscillations (slow waves, spindles, ripples) underlying sleep-related memory consolidation processes, which may have a deleterious effect on patients’ cognition (Gelinas et al., 2016; Lambert et al., 2020; Lambert et al., 2021). In view of the aforementioned evidence that dreams are far from being restricted to REM sleep but also typically occur during light NREM sleep, precisely the stage associated with seizure activation (Minecan et al., 2002), one can expect that nocturnal epileptic activity might have an impact on dreaming processes. This would be more specifically the case for focal seizures. Indeed, epilepsy-induced local dysfunction of the MPFC, the TPO junction, the dlPFC and maybe temporo-mesial structures, could theoretically impact the dreaming process in a quantitative or a qualitative way.

### Focal Seizures and Dreams

The impact of seizures occurring during sleep and their impact on ongoing cognitive processes is still poorly understood. In the same way that stimuli during sleep are likely to be incorporated into dreams, especially if they have a specific intensity, i.e., just below the awakening threshold or a particular meaning for the sleeper (Dement and Wolpert, 1958; Berger, 1963; Koulack, 1969), symptoms related to an epileptic discharge could in theory be incorporated into dream content (this would represent a protective mechanism for sleep according to Freud). This is supported by some clinical observations of patients with nocturnal temporal seizures, reporting that they were having a seizure in their dreams (or reporting subjective feelings associated with seizures as a component of their dream) (Epstein and Hill, 1966; Vercueil, 2005). Very seldom case reports, however, have provided evidence on video-EEG recordings of a concomitant epileptic discharge during sleep (see Box 1). Interestingly, the few documented cases suggest that epilepsy may influence dreaming both in REM sleep (Epstein and Hill, 1966) and NREM sleep (personal case). According to Solms, focal epileptic discharges could also act as intrinsic triggers for the activation of the MPFC yielding an increase in mesocortical dopaminergic activity thought to be involved in limbic structures activation during dreaming (Solms, 2011). Thus, a link between nightmares and nocturnal ictal activity has been suggested (Solms, 1997). This association was explored in a study of 20 patients with temporal lobe epilepsy (TLE) and parasomnia, among which 14 were suffering from recurrent nightmares; dream content included reminiscences of daytime seizures, such as “déjà vu” experiences with intense negative emotions, or feelings of unexplained sense of dread and fear. Interestingly, episodes that could be documented on a video-EEG recording were associated with an ictal discharge; moreover, parasomnia had started coincidentally or after seizure onset and a remission was observed after therapy optimization (Silvestri and Bromfield, 2004).

Interestingly, previously experienced dreams can also be a component of the subjective cognitive content of a diurnal seizure. It may happen notably during temporal lobe seizures, and is referred to as “déjà-rêvé” (Curot et al., 2018). According to the authors, this phenomenon actually includes three distinct entities: (i) the recollection of a specific and detailed dream with a specific date, which is an episodic-like memory; (ii) the reminiscence of a vague dream or elements of dream(s), which is more familiarity-like; (iii) or a “feeling like dreaming” which is a feeling that resembles what happens in dreams. The first two phenomena are mainly elicited by the electric brain stimulation of the medial temporal lobe whereas the “feeling like dreaming” state has less anatomical specificity (Curot et al., 2018). The fact that these evoked memories are unambiguously identified as dream-related raises questions about the neuronal systems involved in dream and diurnal experiences memory; indeed, out of any pathological context, dream memories are rarely confused with semantic or episodic memories from waking life. This suggests that, at the time of encoding or consolidation of the dream memory, a “dream tag” would be either inserted within the memory or that the dream-memory system would be partly distinct from the waking-memory system. Such a dichotomization at the early stages of memory formation would ensure that memories from waking life are not confused with dream memories, which appears to be crucial from an adaptive point of view.

**Box 1. Examples of clinical cases suggesting an influence of epileptic activity on dreaming.**

1. *Concomitant scalp EEG recording was available in the case reported by Epstein and Hill in 1966; in this case, right temporal epileptic rhythmic discharges were observed in the EEG during REM sleep and the patient, who had been awakened by the staff, could report a dream including some of her diurnal seizure symptoms (typical epigastric feeling) within the dream scenario (Epstein and Hill, 1966).*
2. *A 36-year old patient suffering from non-lesional frontal sleep-related hypermotor epilepsy was explored with video-EEG recording. He was interviewed at awakening after an hypermotor seizure which had occurred during late night N2 sleep stage, at 6:30 am. The patient did not report having a seizure, but described being awakened by a nightmare in which he was violently struggling against attackers. Presumably, the hyperkinetic ictal automatisms had been perceived during sleep, and incorporated into the oneiric narrative, suggesting that not only sensory or cognitive experience, but also motor symptoms may become part of the dream scenery (personal case).*

## Memory of Dreams in Epilepsy

### Dreams in Epilepsy Patients

Beyond the observation of a direct association between a single seizure and a particular dream, few studies have specifically investigated dreaming in epilepsy; in general, a mild decrease in dream recall frequency (DRF) is reported in epilepsy patients (Bonanni et al., 2002; Bentes et al., 2011).

#### Dream Recall in Epilepsy Patients

DRF, assessed by a dream diary filled in at home during 60 days, was shown to be higher in patients with focal impaired awareness seizures due to temporal lobe epilepsy (TLE) than in patients with primary generalized seizures (daily DRF: 0.55 ± 0.30 vs. 0.25 ± 0.23, with 85% vs. 61.9% of patients with at least one dream/week, respectively) (Bonanni et al., 2002). Using dream diaries in 52 drug-resistant TLE patients undergoing prolonged video-EEG recordings, another group observed that 71% of patients reported dreams but their DRF was lower than the one of healthy controls completing diaries at home (0.34 ± 0.33 vs. 0.78 ± 0.57 dream/subject/day) (Bentes et al., 2011). In patients with focal impaired awareness seizures, DRF was found to be higher for REM vs. NREM awakenings, in a higher proportion than is usually observed in healthy individuals, suggesting a specific impairment of NREM sleep dream recall in patients; to note, length and structural organization of dreams reported after REM and NREM awakening did not differ (Cipolli et al., 2004).

#### Dream Content in Epilepsy Patients

In a study of Paiva et al. (2011) dream reports in epilepsy patients were shorter and dream content differed from that of control subjects, with a higher proportion of familiar characters and settings, and a lower proportion of success and sexuality. Coherently, in another study of patients experiencing nightmares, short and poorly detailed dream reports have been observed (Silvestri and Bromfield, 2004). Interestingly, some differences between patients were identified according to the laterality of the epilepsy with higher DRF, more aggression, and less animals as well as self-negative expressions in left vs. right TLE (Paiva et al., 2011). Increased vividness and emotionality have also been reported in dreams of epilepsy patients (Gruen et al., 1997). However, complaints of nightmares are only encountered in a small number of patients (Khatami et al., 2006). Finally, some specificities linked to the intrusion of ictal symptoms in dream reports of epilepsy patient have been reported (Epstein, 1964). This observation can be explained by the incorporation of nocturnal epileptic-related symptoms into dreams or by the continuity hypothesis of dreaming. Indeed, in the general population, dream content is strongly influenced by the dreamer’s waking life (Schredl and Hofmann, 2003) and especially by recent and/or emotional events (Vallat et al., 2017a). Interestingly, typical dreams that are widespread in the general population (Schredl et al., 2004) can be very similar to epileptic auras, suggesting the involvement of common brain networks in the two phenomena; this has been described for auras resembling frightening dreams (death, falls, drowning…) or dreams with body image alteration such as the losing-tooth dream (Epstein, 1964, 1967, 2002).

### Factors Involved in Dream Memory in Epilepsy Patients

Data on the effect of the epileptic focus side are conflicting. Some authors reported no influence of epilepsy laterality on DRF (Bonanni et al., 2002; Cipolli et al., 2004) whereas others found lower dream recall in case of right vs. left epileptic focus (Paiva et al., 2011). The presence of a detectable lesion was not found to be associated with DRF, but a higher global cognitive functioning and abstractive abilities were associated with higher DRF and longer dream reports (Bonanni et al., 2002; Cipolli et al., 2004). In the same line, frontal dysfunction in TLE was reported to influence dream content regarding animal content and misfortune (Paiva et al., 2011). Finally, no relationship was found between DRF and the presence of diurnal seizures mentioned on a seizure diary (Bonanni et al., 2002) nor with seizure frequency recorded with video-EEG (Bentes et al., 2011). However, it is important to note that the completeness of the seizure count may have been limited by the subjective report of seizures and by scalp EEG exploration, as some deep-seated brief discharges remain undetectable by surface recordings and, during the night, may manifest only by an arousal (Malow et al., 2000; Peter-Derex et al., 2020).

To our knowledge, the possible relationship between sleep macro- and microstructure, epileptic activity and dream memory in epilepsy patients has never been investigated. The most consistent finding regarding sleep architecture in epilepsy is an increase in wake after sleep onset (Sudbrack-Oliveira et al., 2019), which could be expected to increase DRF (Koulack and Goodenough, 1976). However, no data are available regarding the characteristics of awakenings in epilepsy, although the length and number of awakenings in N2 NREM stage are critical factors for dream recall (Vallat et al., 2017b; Van Wyk et al., 2019). Importantly, long enough intra-sleep awakenings may facilitate dream recall if the content of working memory is not empty (Koulack and Goodenough, 1976). It can be hypothesized that the increased epileptic activity during NREM sleep might alter working memory notably by diminishing its capacity or erasing its content, which could explain the particular decrease in DRF observed upon NREM sleep awakenings in epilepsy patient (Cipolli et al., 2004). Impaired working memory during wakefulness, frequent in epilepsy patients, could also diminish or suppress dream recall at awakening (Arski et al., 2020). REM sleep dreaming would be expected to be less impaired than NREM dreaming in epilepsy patients, given that ictal and inter-ictal epileptic activity is less frequent in REM than in NREM sleep. However some authors reported a decreased REM sleep duration and an increased REM sleep latency in epilepsy patient, particularly in case of nocturnal seizures (Bazil et al., 2000; Scarlatelli-Lima et al., 2016; Mekky et al., 2017). This reduction of REM sleep could participate in the reduced DRF in epilepsy patients. Additionally, changes in density, duration and frequency of sawtooth waves have been reported in TLE patients (Vega-Bermudez et al., 2005); such REM sleep micro-architectural disturbances may also interfere with dreaming processes, given the suspected role of sawtooth waves in driving multifocal fast activities which could be associated with dream content (Siclari et al., 2017; Bernardi et al., 2019; Frauscher et al., 2020).

Several other factors may be involved in a decreased dream recall ability in epilepsy patients, notably anxiety, depression, cognitive disorders and anti-seizure medications which may have an impact on both sleep structure (including arousability) and cognition/dreaming (Bonnet et al., 1979; Kwan and Brodie, 2001; Loring and Meador, 2001; Jain and Glauser, 2014; Keezer et al., 2016; Nicolas and Ruby, 2020). Finally, trait factors that have been identified to covary with DRF and dream content in healthy subjects such as interest in dreams (Schredl and Göritz, 2017), openness to experience (Schredl et al., 2003; Schredl and Göritz, 2017), creativity (Brand et al., 2011), thin personality boundaries (Hartmann, 1989; Schredl et al., 2003), and absorption (Schredl et al., 2003) have not been specifically investigated in epilepsy patients.

## Discussion and Perspective: Current Research Gaps and Potential Future Developments in the Field

Despite the major theoretical interest in the study of dreams as a key component of the sleeping brain’s cognitive activity, few studies have investigated the interactions between dreams and epilepsy. This gap is even more striking as sleep is known to be particularly disrupted in epilepsy and crucial for cognition (Diekelmann and Born, 2010). Most studies on this topic have explored dream recall and dream content in patients with epilepsy (Bonanni et al., 2002; Cipolli et al., 2004; Bentes et al., 2011; Paiva et al., 2011). They have found a moderate decrease in DRF, potentially linked to disturbed sleep with less REM sleep, to impairment of certain cognitive functions and to anti-seizure medications (Bonanni et al., 2002; Bentes et al., 2011). They have also highlighted some characteristics of dream content, being more negative and with more familiar settings, which can be related to patients’ traits or life (altered mood, negative waking life emotions) (Paiva et al., 2011). These results are interesting because they show that epilepsy influences cognition not only during wakefulness but also during sleep, and because they provide information about the cognitive and psychic state of the patients. Although dream function remains a matter of debate, the role of dreams in the regulation of emotions and in the consolidation of memory has been widely emphasized (Malinowski and Horton, 2015; Vallat et al., 2017a; Plailly et al., 2019; Scarpelli et al., 2019). Other theories propose that dreams may act as virtual reality training (to notably improve social and defensive skills) to prepare for waking life challenges (Valli et al., 2005; Hobson, 2009; Hobson et al., 2014; Tuominen et al., 2019) or that they may prevent the brain from overfitting waking experiences by allowing generalization of learned neural representations aiming to ensure a better adaptation to real-world life (Wagner et al., 2004; Hoel, 2021). Altered dreaming processes may thus have consequences on epilepsy patients’ ability to adapt to waking life demands.

Very few projects have addressed the issue of “dreams and epilepsy” from a neurophysiological point of view. Many confounding factors make the study of dream and epilepsy interaction challenging. Longitudinal studies which aim at specifically identifying epileptic activity-related dream modulation would allow to overcome the many biases (underlying epileptogenic condition, comorbidities, treatments) of studies comparing patients and healthy subjects (Putois et al., 2020). A first step could be to more systematically question the epileptic patients explored with video-EEG about their dreams, especially after nocturnal seizures. The use of intracranial EEG investigation may avoid the underestimation of epileptic activity resulting from patients reports or scalp recordings, especially in medial frontal and temporo-parietal epilepsy. Collecting dream narratives in such epilepsy patients, following provoked awakenings after seizures or outside seizures, would allow to assess the direct influence of focal epileptic discharges on dreaming. The occurrence of focal seizures during sleep provides a model of acute transient and spatially limited brain dysfunction that can disrupt or hijack oscillatory interactions within and between networks involved in cognition and notably memory formation (Mendes et al., 2019; Arski et al., 2020). Investigating the effect on dream recall and dream content of epileptic discharges involving the MPFC and/or the TPOJ—but also of other networks engaged in working memory—would advance our understanding of the neurophysiology of dreaming.

As a conclusion, studying the structure of sleep and dream experiences in epilepsy would not only allow to better understand the impact of epilepsy on sleep-associated cognitive functions, but also to explore the neurophysiological substrates of sleep-related cognitive processes from which dreams arise.

## Author Contributions

AC, PR, and LP-D: drafting the manuscript. BF and AV: critical revision of the manuscript. AC, BF, AV, PR, and LP-D: final approval of the version to be published. All authors contributed to the article and approved the submitted version.

## Conflict of Interest

The authors declare that the research was conducted in the absence of any commercial or financial relationships that could be construed as a potential conflict of interest.

## Publisher’s Note

All claims expressed in this article are solely those of the authors and do not necessarily represent those of their affiliated organizations, or those of the publisher, the editors and the reviewers. Any product that may be evaluated in this article, or claim that may be made by its manufacturer, is not guaranteed or endorsed by the publisher.
